# Clinical and ocular factors associated with visual field progression following optic disc haemorrhage in glaucoma

**DOI:** 10.1371/journal.pone.0361865

**Published:** 2026-11-03

**Authors:** Haewon Lee, Ji Eun Song, Seong Ho Kim, Byeong Seok Kim, Yongwoo Lee

**Affiliations:** 1 Kangwon National University School of Medicine, Chuncheon, Republic of Korea; 2 Department of Ophthalmology, Kangwon National University Hospital, Chuncheon, Republic of Korea; 3 Department of Ophthalmology, CHA Bundang Medical Center, CHA University, Seongnam, Republic of Korea; Seirei Hamamatsu General Hospital, JAPAN

## Abstract

**Background:**

To investigate the structural and clinical factors linked to visual field (VF) progression in glaucomatous eyes after the initial detection of optic disc haemorrhage (DH) in patients with primary open-angle glaucoma (POAG).

**Methods:**

This study included 92 eyes of 92 patients who presented with at least one optic DH on stereoscopic optic disc and red-free photography. The included patients had a minimum follow-up of 3 years after initial detection of DH and at least 5 reliable standard automated perimetry investigations. VF progression was assessed using event-based guided progression analysis (GPA). Baseline demographic, clinical, and ocular parameters were examined using Cox proportional hazards regression to identify the independent predictors of functional deterioration.

**Results:**

Among the patients, 32 (34.8%) showed VF progression and 60 (65.2%) remained stable. In univariate analysis, recurrent DH, longer axial length, thinner central corneal thickness (CCT), and intraocular pressure (IOP) fluctuation were significantly associated with VF progression. In multivariate analysis, recurrent DH (HR 4.008, p < 0.001), longer axial length (HR 1.287, p = 0.005), thinner CCT (HR 0.865, p = 0.005), and greater IOP fluctuation (HR 1.997, p = 0.032) remained independently associated with VF progression.

**Conclusions:**

Recurrent DH, longer axial length, thinner CCT, and greater IOP fluctuation were independently associated with VF progression following optic DH. These factors may help identify glaucomatous eyes at higher risk of progression and support closer clinical monitoring after the initial detection of DH.

## Background

Optic disc haemorrhage (DH) is a well-recognised clinical feature of glaucomatous optic neuropathy that has been consistently linked to disease progression [1–4]. Eyes with DH are at an increased risk of further glaucomatous deterioration, suggesting that DH is a crucial prognostic factor for glaucoma [2,5]. Several investigations assessing the temporal relationship between DH and glaucoma progression have suggested that DH often occurs in association with ongoing structural damage to the optic nerve head [4,6–8].

However, not all eyes with DH experience subsequent disease progression, and a considerable proportion of patients maintain a relatively stable clinical course despite DH occurrence [1,2]. Accordingly, the reliability of glaucomatous progression following the first DH detection remains controversial, with variable outcomes reported across studies [1,2,5]. These discrepancies are largely attributable to the variations in study designs, detection modalities, and glaucoma progression definitions. Specifically, multiple longitudinal studies have mainly relied on functional evaluations or conventional imaging methods and have defined follow-up periods based on glaucoma diagnosis rather than the timing of the first detected DH [2,5]. Consequently, the mechanisms underlying the marked inter-individual variability in disease progression following DH remain incompletely understood.

In response to this clinical uncertainty, multiple studies have sought to identify factors associated with glaucoma progression after DH. Shin et al. and De Moraes et al. reported that DH occurring at specific locations is associated with localised structural damage and site-specific disease progression [4,9]. Other studies have reported potential risk factors for progression following DH, including recurrent DH [10], hemodynamic instability such as blood pressure fluctuations [11], focal lamina cribrosa defects [12], and specific morphologic patterns of DH occurring near the optic cup [13,14]. While these studies have provided crucial insights, most have focused on individual risk factors and have not sufficiently explained why only a subset of eyes with DH further develops functional deterioration. Identifying clinical and anatomical markers that differentiate progressors from non-progressors is necessary to optimize monitoring frequency and treatment intensity. Although different individual risk factors have been proposed, few longitudinal studies have systematically integrated the structural and clinical parameters to determine their combined influence on subsequent progression.

Thus, this study aimed to examine the structural and clinical factors associated with visual field (VF) progression in eyes with glaucoma after initial detection of DH. We sought to determine which parameters reliably predicted subsequent functional deterioration by evaluating ocular and clinical variables, potentially facilitating personalised management strategies for patients with DH.

## Methods

The Institutional Review Board of the Kangwon National University Hospital approved this study (KNUH-2025-09-004). The requirement for informed consent was waived due to the retrospective nature of the study. All the study procedures adhered to the tenets of the Declaration of Helsinki.

### Subjects

The medical records of patients diagnosed with primary open-angle glaucoma who visited the glaucoma clinic at Kangwon National University Hospital between 1 January 2019 and 1 December 2020 were retrospectively reviewed. The authors had access to the medical records and clinical data for research purposes from 12 September 2025–31 December 2025. To be included in this study, patients were required to have (1) one or more identified optic DH on stereoscopic optic disc photography (SDP) and red-free photography (RFP), (2) a follow-up period of at least 3 years following DH with regular follow-up visits at 6-month intervals, and (3) at least five reliable standard automated perimetry results following DH occurrence. For patients in whom both eyes fulfilled the inclusion criteria, only one eye was included in the primary analysis to avoid inter-eye correlation. Among bilateral eligible eyes, the eye with the earlier first-detected optic disc haemorrhage was selected according to a pre-specified rule. All patients were followed up with regular disc photography, RNFL thickness measurement, and VF examination. Demographic data (age and sex) were recorded for all included patients, and the age was calculated at the time of DH occurrence. Furthermore, family history of glaucoma, systemic comorbidities including hypertension, diabetes mellitus, and the presence of cold extremities or migraines were documented. We excluded patients with best-corrected visual acuity worse than 20/40, spherical equivalent (SE) outside the range of −6.0 to +3.0 diopters (D) (thereby excluding eyes with high myopia [SE <−6.0 D]), significant media opacity affecting test reliability, history of ocular surgery other than uncomplicated cataract surgery or ocular trauma, concomitant optic neuropathies or retinal diseases that might affect VF, or unreliable VF examinations.

### Ophthalmic examinations

Routine glaucoma assessments included the measurement of visual acuity, refraction, slit-lamp examination, intraocular pressure (IOP) using Goldmann applanation tonometry, SE, and dilated stereoscopic optic disc evaluation. SDP and RFP (Visucam; Carl Zeiss Meditec AG, Jena, Germany) were used for DH evaluation. All photographs were independently reviewed by two experienced ophthalmologists (SHK and BSK), who were masked to patient identity, clinical characteristics, and longitudinal VF outcomes. Disagreements regarding the presence or recurrence of DH were resolved by consensus with a third glaucoma specialist (JES). Recurrent DH was defined as a newly detected haemorrhage after complete resolution of the previous haemorrhage during follow-up. RNFL thickness and optic nerve head parameters were assessed using spectral-domain optical coherence tomography (Spectralis OCT; Heidelberg Engineering, Heidelberg, Germany). Visual function was evaluated using standard automated perimetry (Humphrey Visual Field Analyzer HVF II; Carl Zeiss Meditec Inc., Dublin, OH, USA). Axial length and anterior chamber depth (ACD) were measured using IOL Master 500 (Carl Zeiss Meditec, Dublin, CA, USA). Central corneal thickness (CCT) was measured using ultrasound pachymetry. Additionally, DH recurrence and IOP fluctuations were evaluated during follow-up. Baseline parameters were evaluated at the time of first DH detection. IOP fluctuation was defined as the standard deviation (SD) of the IOP until the first treatment intensification (addition or change of glaucoma medication or glaucoma surgery) to minimize the influence of treatment modification on IOP variability. In eyes that did not undergo treatment modification during follow-up, all IOP measurements until the last follow-up visit were included.

### Visual field analysis and progression

This study included only subjects who provided reliable VF results with reliability indices maintained at a fixation loss ≤ 20% and false negatives/positives ≤ 15%. A glaucomatous VF defect was identified based on at least one of the following criteria: (1) a Glaucoma Hemifield Test (GHT) result categorised as “outside normal limits,” (2) a cluster of three or more points on the pattern deviation plot with P < 1%, or (3) a pattern standard deviation (PSD) with P < 5%. VF progression was assessed using event-based analysis with the guided progression analysis (GPA) software, where “likely progression” was defined as a significant decline at three or more test points on three consecutive examinations. According to the Humphrey GPA algorithm, the first two reliable visual field examinations were used as the baseline for progression analysis. For time-to-event analysis, the event date was defined as the date on which likely progression was first confirmed. Eyes that did not meet these criteria were classified as non-progressors and were censored at the date of the last reliable VF examination.

### Statistical analysis

Statistical analyses were performed using SPSS Statistics version 28.0 (IBM Corp., Armonk, NY, USA). Continuous data were presented as mean ± SD, and categorical data were reported as frequencies and percentages. Independent t-tests for continuous variables and chi-square tests or Fisher’s exact tests for categorical variables were used to compare the baseline characteristics between the progressor and non-progressor groups.

Cox proportional hazards regression was conducted to identify factors independently associated with VF progression. Time to VF progression was defined as the interval between the first detected DH and the first VF examination demonstrating progression according to GPA, whereas eyes without progression were censored at the last reliable VF examination. Variables with *P* < 0.10 in the univariate analysis were included in the multivariable model. Multicollinearity among the variables included in the multivariable model was assessed using variance inflation factors, and no evidence of significant multicollinearity was identified. The results are presented as hazard ratios (HRs) with 95% confidence intervals. To improve the clinical interpretability of the effect estimates, CCT was analyzed per 10-μm increment. Statistical significance was set at *P* < 0.05.

## Results

A total of 118 eyes were screened for this study. Of these, 23 eyes were excluded because of poor image quality or low-test reliability (n = 15), missing ophthalmic examinations (n = 5), or high myopia (n = 3). Among the remaining 95 eligible eyes, three patients contributed both eyes. To avoid inter-eye correlation, only one eye per patient was included according to a pre-specified selection rule. Consequently, 92 eyes from 92 patients were included in the final analysis. During the follow-up period, VF progression was identified in 32 eyes (34.8%), and 60 eyes (65.2%) remained stable.

The mean age of the patients was 61.33 ± 15.53 years, with a female predominance (53.7%). Systemic comorbidities included hypertension (29.3%), diabetes mellitus (19.6%), cold extremities (31.5%), migraines (19.6%), and a family history of glaucoma (13%). Regarding ocular characteristics, the mean spherical equivalent and axial length were −2.35 ± 3.61 D and 24.74 ± 1.78 mm, respectively. The average CCT was 539.29 ± 38.41 μm, and the ACD was 2.93 ± 0.73 mm. Baseline structural and functional indices revealed an 80.98 ± 16.25 μm global RNFL thickness and a −5.62 ± 6.11 dB VF mean deviation (MD). Additionally, the mean IOP fluctuations was 1.50 ± 0.63 mmHg, and recurrent DH occurred in 20.7% of patients (Table 1). There was no significant difference in the number of glaucoma medications at baseline between the two groups. However, medication intensification and glaucoma surgery during follow-up were more frequently performed in eyes with VF progression (S1 Table). The mean follow-up duration was 60.50 ± 9.43 months in the progression group and 61.61 ± 8.11 months in the non-progression group, with no significant difference between the groups (*P* = 0.342).

**Table 1:** Baseline characteristics of glaucomatous eyes with disc haemorrhage.

Variable	Total (n = 92 eyes from 92 patients)
Age, years	61.32 ± 15.52
Female, n (%)	51 (55.4)
Family history of glaucoma, n (%)	13 (14.1)
Hypertension, n (%)	27 (29.3)
Diabetes mellitus, n (%)	18 (19.6)
Cold extremities, n (%)	29 (31.5)
Migraine, n (%)	18 (19.6)
Recurrence of DH, n (%)	19 (20.7)
SE, D	-2.35 ± 3.61
AXL, mm	24.74 ± 1.78
ACD, mm	2.93 ± 0.73
CCT, μm	539.29 ± 38.41
Baseline IOP, mmHg	13.15 ± 3.27
Baseline global RNFLT, μm	80.98 ± 16.25
Baseline global MRW, μm	208.27 ± 57.90
Baseline MD, dB	-5.62 ± 6.11
Baseline PSD, dB	5.09 ± 3.75
IOP fluctuation, mmHg	1.50 ± 0.63

Data are presented as mean ± standard deviation or n (%).

DH = disc haemorrhage; SE = spherical equivalent; AXL = axial length; ACD = anterior chamber depth; CCT = central corneal thickness; IOP = intraocular pressure; RNFLT = retinal nerve fiber layer thickness; MRW = minimum rim width; MD = mean deviation; PSD = pattern standard deviation; D = diopters; dB = decibel

Table 2 shows the results of the univariate and multivariate Cox proportional hazards regression analyses for factors associated with VF progression after the first detected DH. In the univariate analysis, recurrence of DH, longer axial length, thinner CCT (per 10-μm increase), and greater IOP fluctuation (per 1-mmHg increase in SD) were significantly associated with VF progression. In the multivariate analysis, recurrence of DH (HR, 4.008; *P* < 0.001), longer axial length (HR, 1.287; *P* = 0.005), thinner CCT (HR, 0.865 per 10-μm increase; *P* = 0.005), and greater IOP fluctuation (HR, 1.997 per 1-mmHg increase; *P* = 0.032) remained independently associated with VF progression after adjustment for potential confounding factors.

**Table 2:** Cox proportional hazards regression analysis of factors associated with visual field progression in glaucoma eyes with disc haemorrhage.

	Univariate			Multivariate		
	B (S.E)	HR (95% CI)	P value	B (S.E)	HR (95% CI)	P value
Age, years	−0.012 (0.011)	0.987 (0.966-1.008)	0.226			
Female gender, n	−0.535 (0.356)	0.585 (0.291-1.177)	0.133			
Family history of glaucoma, n	0.249 (0.487)	1.282 (0.494-3.332)	0.610			
Hypertension, n	−0.565 (0.428)	0.568 (0.246-1.314)	0.186			
Diabetes mellitus, n	−0.408 (0.487)	0.665 (0.256-1.727)	0.402			
Cold extremities, n	−0.581 (0.428)	0.560 (0.242-1.294)	0.175			
Migraine, n	0.218 (0.428)	1.244 (0.538-2.876)	0.610			
Recurrence of DH, n	**1.306 (0.358)**	**3.690 (1.829-7.442)**	**<0.001**	**1.388 (0.382)**	**4.008 (1.896-8.475)**	**<0.001**
SE, D	−0.047 (0.044)	0.281 (0.875-1.040)	0.281			
AXL, per 1 mm increase	**0.233 (0.090)**	**1.262 (1.058-1.505)**	**0.010**	**0.252 (0.090)**	**1.287 (1.078-1.537)**	**0.005**
ACD, mm	−0.144 (0.284)	0.611 (0.496-1.510)	0.611			
CCT, per 10μm increase	**−0.094 (0.046)**	**0.910 (0.832-0.996)**	**0.041**	**−0.145 (0.051)**	**0.865 (0.783-0.957)**	**0.005**
Baseline IOP, mmHg	0.035 (0.055)	1.036 (0.930-1.154)	0.523			
Baseline global RNFLT, μm	−0.019 (0.012)	0.981 (0.959-1.004)	0.102			
Baseline global MRW, μm	0.000 (0.005)	1.000 (0.989-1.010)	0.966			
Baseline MD, dB	0.009 (0.040)	1.009 (0.932-1.092)	0.822			
Baseline PSD, dB	0.070 (0.058)	1.072 (0.957-1.201)	0.229			
IOP fluctuation, per 1 mmHg increase	**0.563 (0.282)**	**1.757 (1.011-3.052)**	**0.046**	**0.692 (0.323)**	**1.997 (1.060-3.764)**	**0.032**

B = beta coefficient; S.E = standard error; HR = hazard ratio; CI = confidence interval; DH = disc haemorrhage; SE = spherical equivalent; AXL = axial length; ACD = anterior chamber depth; CCT = central corneal thickness; IOP = intraocular pressure; RNFLT = retinal nerve fiber layer thickness; MRW = minimum rim width; MD = mean deviation; PSD = pattern standard deviation; D = diopters; dB = decibel

*P* values in boldface indicate statistical significance.

Figs 1 and 2 show the representative cases illustrating the contrast between eyes with and without VF progression. The eye demonstrating progression was characterised by a longer axial length (25.92 mm) and a thinner CCT (503 μm), with serial VF tests confirming functional deterioration (Fig 1). Contrastingly, the eye without progression, which featured a relatively shorter axial length (23.76 mm) and thicker CCT (534 μm), maintained a stable optic disc appearance and VF parameters throughout the follow-up period (Fig 2).

**Fig 1:**
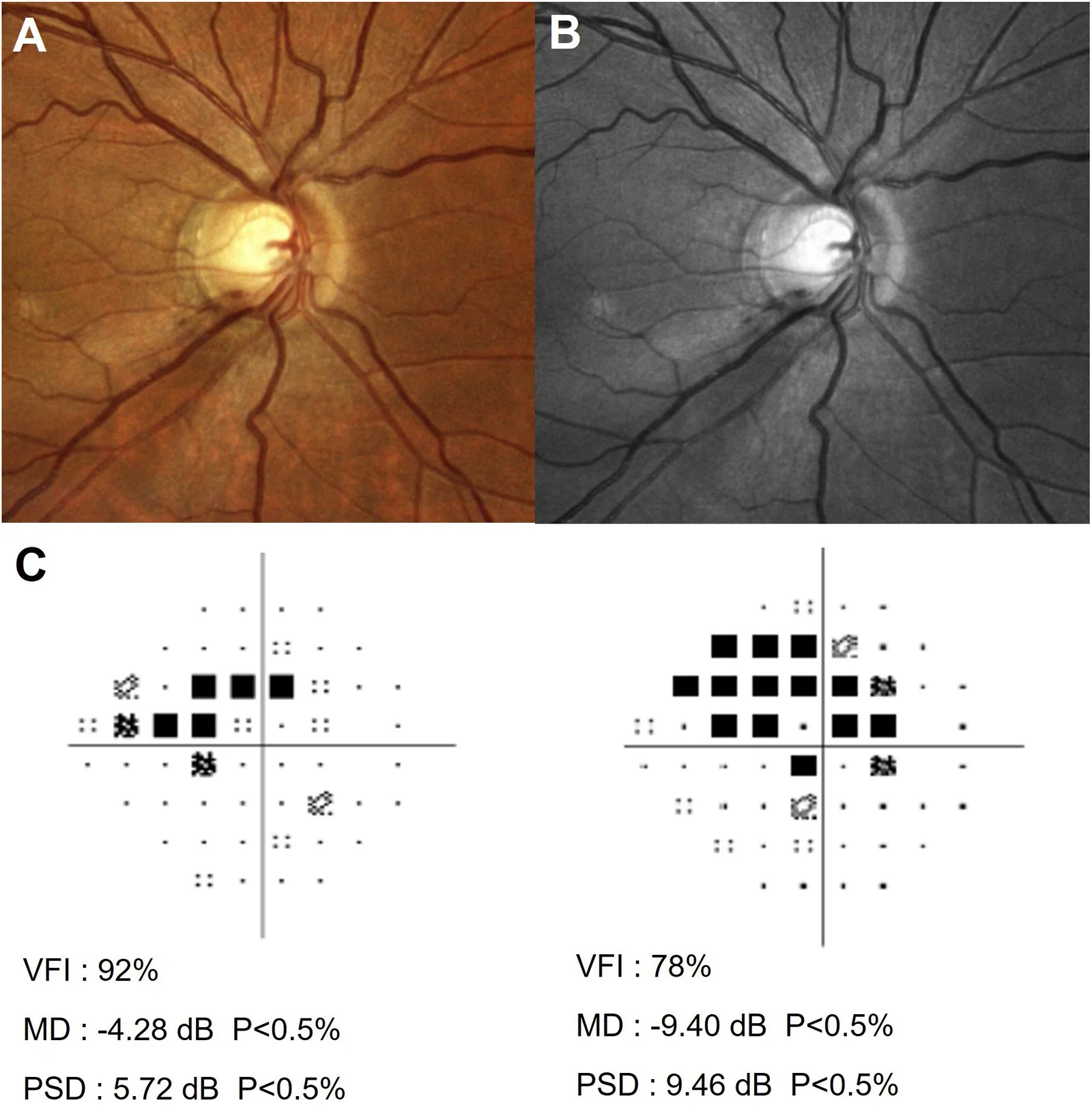
Representative case with visual field progression following optic disc haemorrhage (DH). (A) Optic disc photography and (B) red-free photography obtained at the time of first-detected DH. (C) Visual field tests at baseline (left) and 3 years later (right), showing progression.

**Fig 2:**
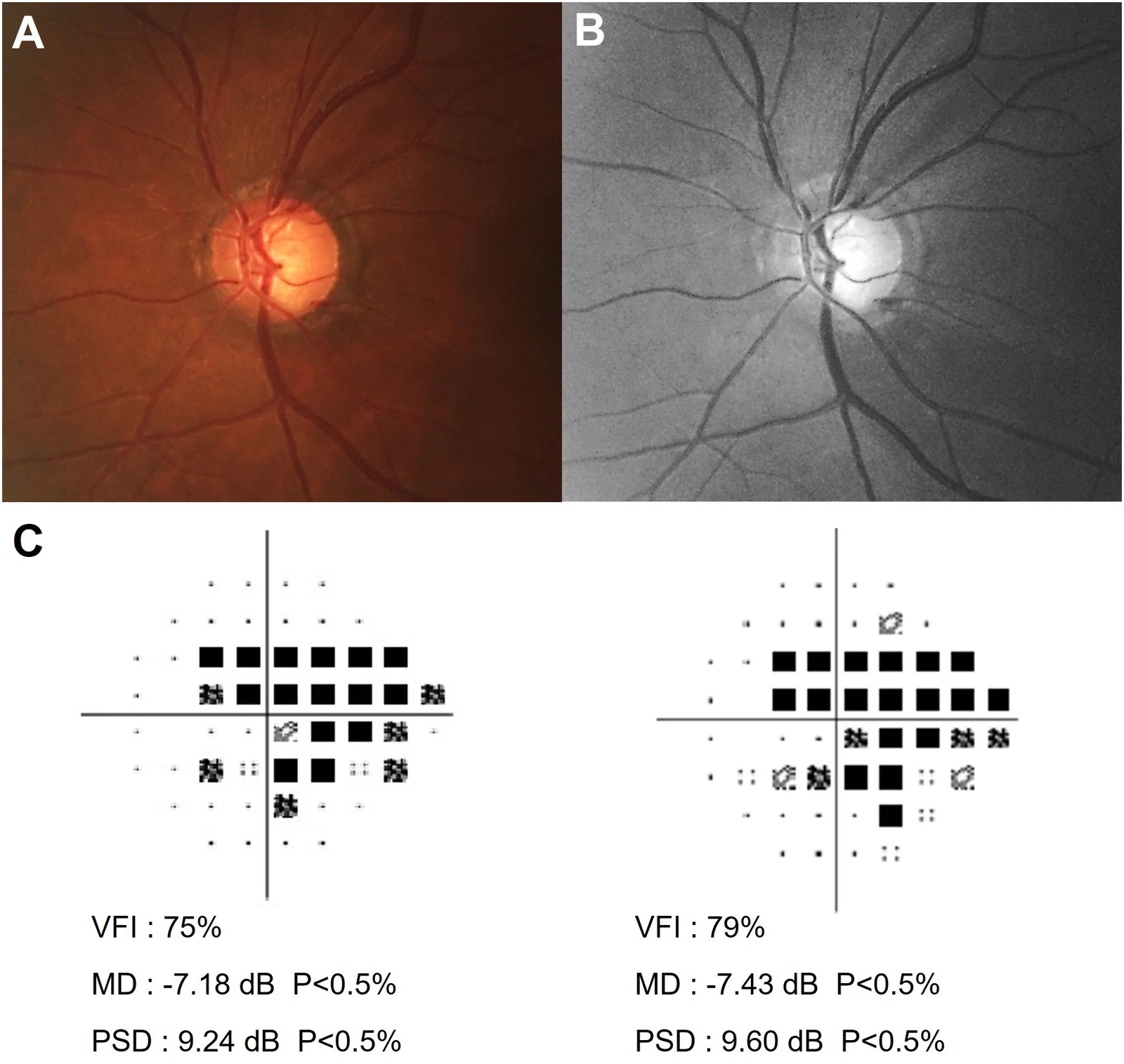
Representative case without visual field progression following optic disc haemorrhage (DH). (A) Optic disc photography and (B) red-free photography obtained at the time of first-detected DH. (C) Visual field tests at baseline (left) and 3 years later (right), showing stable visual field findings without progression.

## Discussion

This study examined factors associated with VF progression in glaucomatous eyes with DH. Although DH is a well-established risk factor for glaucoma progression, the clinical outcomes differ among patients. Herein, longer axial length, thinner CCT, and greater IOP fluctuation were independently associated with progression in eyes with DH. Because axial length and CCT are surrogate clinical markers rather than direct measures of biomechanical properties, the underlying mechanisms should be interpreted with caution. Thus, DH may represent a clinical event that identifies eyes with increased susceptibility to glaucomatous progression rather than serving solely as an independent predictor of disease progression.

In the present analysis, a longer axial length and thinner CCT were markedly linked to VF progression. These two parameters may be interpreted as surrogate clinical markers of structural characteristics related to the biomechanical susceptibility of the optic nerve head rather than direct measures of ocular biomechanics. In myopic eyes, the posterior sclera is thinner, and the lamina cribrosa is often stretched and flattened [15–17]. This structural change compromises the ability of the lamina cribrosa to withstand translaminar pressure gradients [18]. Consequently, an elongated eye with a mechanically vulnerable lamina cribrosa is less capable of recovering from the insult when DH signals a microvascular breakdown or mechanical failure of the neuroretinal rim, leading to axonal death and functional progression [19,20]. As expected, axial length was strongly correlated with spherical equivalent (r = −0.77, *P* < 0.001). Given that axial length more directly reflects ocular structural characteristics than refractive error, axial length was selected for the multivariable analysis. Similarly, a thinner CCT correlates with a greater deformity of the lamina cribrosa and increased vulnerability of the optic nerve head, beyond being merely a source of tonometric errors [14,21]. Patients with thinner corneas exhibit lower mechanical stiffness of the lamina cribrosa and are more likely to experience greater tissue deformation under the same IOP elevation [14]. Our findings indicate that eyes with compromised biomechanical support could be more susceptible to irreversible damage after ischemic or mechanical stress, as indicated by DH. However, it should be acknowledged that axial length and CCT are indirect proxies of biomechanical vulnerability; direct measurements such as corneal hysteresis, ocular response analyser-derived metrics, and lamina cribrosa deformation indices were not available in the present dataset, and future studies incorporating these parameters are warranted.

Although baseline IOP was not identified as a significant risk factor, IOP fluctuations showed a strong association with VF progression. The role of IOP fluctuations in glaucoma progression has been debated, with inconsistent results across studies [22–25]. Our findings suggest that dynamic pressure instability acts as a crucial modifier, rather than a primary driver, of glaucomatous damage. Repeated short-term IOP elevation imposes cyclic mechanical strain on the optic nerve head, accelerating axonal injury via direct mechanical stress and disruption of axonal transport [18–20,26]. Given these mechanisms, dynamic stress may exceed biomechanical tolerance in glaucoma patients with DH, who are likely already to have focal structural weakening, providing a plausible explanation for why some eyes rapidly progress following DH, while others remain stable.

A crucial distinction between this study and previous investigations is the technique used to evaluate disease progression. Several studies have reported that RNFL progression defects occur in a considerable proportion of patients following DH [2,27–29]. In contrast, the present study assessed progression based on functional criteria using VF testing and noted a VF progression rate of 34.8%. This difference is consistent with the natural course of glaucoma in which structural damage generally precedes functional loss [30–32]. Despite applying stringent functional criteria for progression, the axial length, CCT, and IOP fluctuations remained independent predictors, indicating that these biomechanical and physiological factors directly contribute to clinically meaningful visual function loss. However, structural and functional predictors were not directly compared within the same model; future studies should assess whether structural parameters provide additional predictive value over functional measures. Additionally, recurrent DH was also independently associated with subsequent VF progression in the multivariable analysis. This finding is consistent with previous studies [28,33] suggesting that recurrent DH reflects ongoing glaucomatous instability and is associated with an increased risk of disease progression. Repeated episodes of DH may indicate persistent structural or vascular vulnerability at the optic nerve head, identifying eyes that require closer monitoring and more intensive clinical management.

This study has several limitations. First, this study was limited by its retrospective design and relatively small sample size, which may have restricted definitive causal inferences and statistical power. Although rigorous inclusion criteria and longitudinal follow-up were applied, the events-per-variable ratio (EPV) in this study was below the recommended threshold of 10, which may have reduced the precision and stability of the estimated hazard ratios. Therefore, these findings should be interpreted with caution and warrant confirmation in larger prospective studies. Second, other potentially relevant factors, such as microvascular dropout (MvD), were not assessed because of the unavailability of OCT angiography at our centre during the study period. Future longitudinal studies incorporating MvD are required to comprehensively elucidate the complex interplay between biomechanical susceptibility and microvascular impairment in the eyes with DH. Third, IOP fluctuations were evaluated based on clinic visits rather than 24-hour monitoring, which may not capture nocturnal spikes or short-term variability. Nevertheless, visit-to-visit IOP fluctuations have consistently been shown to be potent predictors of long-term VF deterioration. Forth, this study did not evaluate DH location or morphological characteristics. Prior investigations indicate that DH occurring at specific sectors of the optic disc may be associated with localized structural damage and significant VF deterioration, and future studies incorporating these features are warranted. Furthermore, structural and functional predictors were not directly compared within the same analytical model. Future studies integrating both structural and functional outcomes are needed to determine their relative and complementary prognostic value. Lastly, because eyes with high myopia (SE <−6.0 D) were excluded, the present findings regarding axial length should be interpreted within the range of non-highly myopic and moderately myopic glaucomatous eyes. The applicability of these findings to highly myopic glaucoma requires further validation in future studies.

This study suggests that structural and clinical factors, including longer axial length, thinner CCT, and greater IOP fluctuation, are associated with VF progression after DH in glaucoma. Rather than serving as a uniform predictor of progression, DH may represent a clinically important event that identifies eyes at greater risk of subsequent functional deterioration. These results provide a practical framework for risk stratification and individualised management of glaucomatous eyes after DH detection.

## Supporting information

S1 Table Glaucoma treatment after the first-detected disc haemorrhage. (DOCX)
