# Supplementary material for: Clinical and ocular factors associated with visual field progression following optic disc haemorrhage in glaucoma
Source: PLoS One. 2026 Nov 3;21(11):e0361865. doi: 10.1371/journal.pone.0361865 (PMC13730382; doi:10.1371/journal.pone.0361865)
Supplement: Supplementary file 1 [file pone.0361865.s001.docx]

**S1 Table. Glaucoma treatment after the first-detected disc haemorrhage**

| **Variable** | **VF Progression**  **(n=32)** | **VF non-progression (n=60)** | **P value** |
| --- | --- | --- | --- |
| Number of glaucoma medications at baseline | 0.82 ± 0.58 | 0.65 ± 0.59 | 0.211 |
| Medication intensification during follow-up, n (%) | 17 (53.1) | 9 (15) | **<0.001** |
| Glaucoma surgery  during follow-up, n (%) | 4 (12.5) | 0 (0) | **0.004** |

VF = visual field

*P* values in boldface indicate statistical significance.
